# The effect of physician density on colorectal cancer stage at diagnosis: causal inference methods for spatial data applied on regional-level data

**DOI:** 10.1186/s12942-023-00323-w

**Published:** 2023-01-19

**Authors:** Dajana Draganic, Knut Reidar Wangen

**Affiliations:** Department of Health Management and Health Economics, Institute of Health and Society, University of Oslo, Oslo, Norway

**Keywords:** Colorectal cancer, Preventive services, General practitioners, Specialists, Regional variations, Physician density, Causal methods for spatial data, Bayesian statistics

## Abstract

**Background:**

The early detection of colorectal cancer (CRC) through regular screening decreases its incidence and mortality rates and improves survival rates. Norway has an extremely high percentage of CRC cases diagnosed at late stages, with large variations across municipalities and hospital catchment areas. This study examined whether the availability of physicians related to CRC primary diagnosis and preoperative investigations, or physician density, contributes to the observed geographical differences in late-stage incidence rates.

**Method:**

Municipality-level data on CRC stage at diagnosis were obtained from the Cancer Registry of Norway for the period 2012–2020. Physician density was calculated as the number of physicians related to CRC investigations, general practitioners (GPs) and specialists per 10,000 people, using physician counts per municipality and hospital areas from Statistics Norway. The relationship was examined using a novel causal inference method for spatial data—neighbourhood adjustment method via spatial smoothing (NA approach)—which allowed for studying the region-level effect of physician supply on CRC outcome by using spatially referenced data and still providing causal relationships.

**Results:**

According to the NA approach, an increase in one general practitioner per 10,000 people will result in a 3.6% (CI −0.064 to −0.008) decrease in late-stage CRC rates. For specialists, there was no evidence of a significant correlation with late-stage CRC distribution, while for both groups, GPs and specialists combined, an increase of 1 physician per 10,000 people would be equal to an average decrease in late-stage incidence rates by 2.79% (CI −0.055 to −0.001).

**Conclusion:**

The study confirmed previous findings that an increase in GP supply will significantly improve CRC outcomes. In contrast to previous research, this study identified the importance of accessibility to both groups of physicians—GPs and specialists. If GPs encounter insufficient workforces in hospitals and long delays in colonoscopy scheduling, they will less often recommend colonoscopy examinations to patients. This study also highlighted the efficiency of the novel methodology for spatially referenced data, which allowed us to study the effect of physician density on cancer outcomes within a causal inference framework.

**Supplementary Information:**

The online version contains supplementary material available at 10.1186/s12942-023-00323-w.

## Introduction

Colorectal cancer (CRC) is one of the most avoidable and preventable of all types of cancer, with a 90% 5 year survival rate for cases not discovered in later stages when the cancer has spread from the colon and rectum. Timely screening is one of the most important mechanisms for preventing late-stage diagnosis and improving overall CRC outcomes through the early detection and removal of precancerous polyps [1–3]. Despite the benefits of regular CRC screening, screening tests are performed less frequently than that for other types of cancers [4]. Previous studies have reported that, among other factors, area of residence could be a strong predictor of patients not undergoing frequent and timely CRC screenings. Studies from Australia and U.S. showed that rural residents have a longer interval from the first symptoms to the screening test than urban residents, with the diagnostic interval contributing the most to delayed diagnosis [5, 6]. Different access to primary care, diagnostic, and specialist services between rural and urban residences may have an important effect on prolonging help-seeking and the diagnostic interval. The centralisation of screening services in urban areas makes these services inaccessible to remote areas and increases travelling time and costs, which are important factors in individuals’ decisions to participate in screenings, according to a Norwegian study [7]. Furthermore, the mentioned study from Australia showed that GPs in remote areas, having lower numbers of gastroenterologists, surgeons, and other specialists on disposal, could exert a higher threshold before making a decision on sending a referral for screening [6]. In places where screening programmes are not addressed at the national level and there is no direct access to specialist care, primary care physicians’ recommendations are extremely important. Considering these facts, in the present study, we addressed the following question: Will the increase in physician’s regional availability (both primary and specialists) reduce the disparities of diagnostic outcomes and increase the probability of detecting CRC cancers in the early phases?

This question has been addressed by several previous studies. Roetzheim et al. [4] investigated the impact of physician density across counties in Florida, U.S., and found that a 10% increase in physician supply will affect a 5% decrease in late-stage CRC diagnosis. Ananthakrishnan et al. [8], using county data from Pennsylvania, U.S., reported a negative association between late-stage CRC and the density of primary care physicians (PCP) or gastroenterologists but not for these two groups combined. Their study claimed that higher physician density could improve access to healthcare by decreasing waiting times and travel costs and increasing the chances of patients visiting doctors. In addition, higher availability could increase competition among physicians and improve the quality of services. Patients would have more options to choose among physicians who differ with respect to their skills and qualities. Blair & Datta [9] did not observe any significant differences in physician density across Canadian provinces.

Several studies have used aggregate-level data and were conducted as ecological studies, the main limitation of which is the establishment of a causal relationship [4, 8]. To confirm their results, there is an apparent need for further follow-up studies conducted at the individual level, while the exploratory nature of selecting variables may also increase the risk of falsely concluding that an association exists, even if it occurred by chance. Despite this limitation, such data were found to be more convenient for investigating this topic. The main variable of interest, availability of physicians, is more valid if measured over a certain geographic area [8]. Blair and Datta [9] used individual-level CRC case data and linked these data with physician density data using the postal codes of patients’ residences. The study was performed as a cross-sectional study, and thus, it was not possible to establish causality [9]. When matching patient- and area-level data, it is challenging to match different registries and capture patients’ movements. This is because they most likely belong to different GPs or hospitals not necessarily linked to their place of residence which changes over time due to their migrations across referral regions.

This study investigated late-stage CRC disparities among Norwegian municipalities that are attributable to geographic disparities in healthcare access using municipality-level data. Our study extends current research on this topic by applying advanced statistical methods in spatial epidemiology [10]. Specifically, we applied a neighbourhood adjustment method (NA method) involving a spatial smoothing, which allowed us to use spatially referenced data to establish causal relationships. In addition, we compared these estimates with estimates obtained from standard methods with spatial data which are, in contrast, only estimated correlations (Besag, York, and Mollié [BYM] method).

We collected datasets with information on the incidence rates and other available and relevant socioeconomic and demographic covariates at the municipality level. However, even if we had included different relevant covariates, difficult-to-capture confounders could still have posed a critical threat, and if we had used structural data, the threat could have occurred at different levels. As we examined the region-level effect on disease outcomes, we were especially interested in adjusting our estimates for place-specific drivers or unobserved variables on region-level [11]. The NA approach was developed to adjust for all potentially unobserved regional variables or spatial confounders [12]. The approach starts with the assumption that these variables are expected to be spatially correlated and that nearby municipalities are expected to have similar values for such confounders.

We leveraged rich registry data from Norway to address this question. In addition, we chose this study area because it allowed us to directly explore the effect of the area of residence on health disparities. Despite having a higher number of doctors per 10,000 people compared to other EU countries, Norway faces an uneven geographical distribution of general physicians and shortages of physicians in some areas [13]. There is also a shortage of medical staff involved in the screening procedures. As they perform many tasks in hospitals other than colonoscopies, these specialists are not easily recruited from smaller hospitals. However, Norway has relatively decentralised services and universal health coverage with equal access.

## Methods

### Data and measures

CRC cases that occurred between 2012 and 2020 in Norway were included in the study (n = 38,968), with available data on staging (approximately 90% of the total data). CRC incidence rates were obtained from the Cancer Registry of Norway and corresponded to the yearly number of new cases of colorectal tumours observed at the municipality level. The municipality (*kommune*) is the lowest level administrative division in Norway. As the municipalities in Norway are undergoing continuous consolidation, data were adjusted based on the latest administrative changes from 2020, according to which Norway is divided into 356 administrative regions. Cases were drawn using the third edition of the International Classification of Diseases for Oncology codes for colon and rectal cancers (C18.0–18.9, C19.9, C26.0, C20.9). According to Surveillance, Epidemiology, and End Result Program stages were classified as localised (referred to as the early stage in this paper) and regional, and distant (referred to as the late stage).

To evaluate access to healthcare, we chose municipality-level measures of the availability of physicians’ resources for 2011 obtained from Statistics Norway (Municipality Healthcare Services). All physicians involved in the primary diagnostic and preoperative investigations were included in the study such as GPs, gastroenterologists, surgeons, diagnostic radiologists, and pathologists.

There were two primary outcome variables of interest in this study. These variables included: (1) CRC stage at diagnosis based on the SEER summary stage classified as late-stage (if regional or distant) and early stage (if localised) and (2) relative risk or standardised incidence ratio.

Municipality-level relative risk rates for late-stage CRC were calculated as the ratio between the number of observed and expected cases. Expected cases per municipality were equal to the expected counts of cases in the observed area after applying rates that are specific to Norway for the particular age and gender groups. Specifically, we calculated country-level disease rates for four age-sex strata (females 0–74 years, females 75 years and older, males 0–74 years, males 75 years and older) and applied them to each of the observed areas based on their age-sex specific population structure in the reported period. A relative risk of 1 indicated that the number of observed cancer cases in that municipality was equal to the number of cancer cases expected for the entire population. Relative risks greater than 1 indicated that more cases occurred than expected, and relative risks lower than 1 indicated that fewer cases occurred than expected.

Key exposure was the density of municipality-level physicians per 10,000 people. We expressed physician density as physician-per-population-ratio or number of physicians per 10,000 people at the municipality level. The physicians included GP counts per municipality, as well as specialist counts that were closely related to the CRC diagnostic and preoperative investigations, such as gastroenterologists, surgeons, diagnostic radiologists, and pathologists.

Other covariate data included socioeconomic, lifestyle, and urban–rural population information and additional available information, including colonoscopy rates. Municipality-level socioeconomic status was assessed based on the following variables: percentages of the population living in urban areas, the population living in households with persistent low income, and the population not employed or studying. Municipality-level lifestyle data included variables on smoking status and exercise levels in the population. Most of the data were obtained from Statistics Norway (2022) or the Norwegian Institute for Public Health (2022). In addition, number of colonoscopies per 10,000 people was obtained from the Gastronet project (a network for quality assurance of gastroenterology in Norway) at the Cancer Registry of Norway.

### Statistical analysis

The main goal of our analysis was to estimate the causal effect of physician density on late-stage CRC distribution. We considered two analyses to investigate the relationship of interest in the presence of unmeasured spatial confounders [12]. We investigated this relationship by applying a novel causal inference method for spatial data, that is, a neighbourhood adjustment model via spatial smoothing, which we referred to as the NA approach. We compared results from the NA approach to results from the more established Bayesian hierarchical BYM model for count data, which we referred to as the BYM approach. This approach only estimates correlations [14].In the BYM approach, we modelled late-stage CRC incidence as a Poisson-distributed outcome using a log link with exposure (physician density) and other available covariates (Table 1). This approach included two random effects: a spatially structured random effect, which smooths locally towards the values of nearby areas, and an unstructured random effect that smooths globally, towards the overall average. We assumed that spatially correlated random effects follow the CAR distribution. The CAR prior was defined by an adjacency matrix, where geographically adjacent neighbours were given a value of 1, and all other pairs, a value of 0.

**Table 1:** Municipality-level measures

Variable	Median (Q1–Q3)
Number of GPs per 10,000 people^a,d^	0.21 (0.17–0.32)
Number of specialists closely related to CRC diagnosis per 10,000 people^b,d^	1.43 (1.07–1.66)
Number of colonoscopies per 10,000 people^b,d^	9.6 (8.4–12)
Area and population of urban settlements^a^	660 (2400–8100)
Total population^a^	5200 (2200–13000)
Percentage of people over 65 years old^a,d^	0.18 (0.15–0.20)
Median income (× 1000)^c^	530 (500–580)
Not employed or studying (percentage)^c^	0.18 (0.16–0.21)
Exercising less frequently then weekly(percentage)^c^	0.26 (0.22–0.31)

^a^Statistics Norway

^b^Cancer registry of Norway (“Gastronet” project)

^c^Municipal health statistic bank

^d^Authors' calculations

However, Paciorek [15] found that simply accounting for spatial correlation may not fully resolve spatial confounding and that commonly used estimators, including the BYM approach, can be biased in the presence of unmeasured spatial confounding. As a response to these findings, Schnell and Papadogeorgou [12] proposed the NA method that adjusts for unobserved spatial confounders by blocking their statistical dependence on either the treatment or the exposure variable. Specifically, in the NA approach, we addressed confounding bias by joint spatial modelling of exposure and unobserved spatial confounders. The bias, denoted as $B(X)$, is a result of attributing the effect of the confounder on the outcome to the exposure variable when exposure and spatial confounders (U) are correlated [10]. The bias was modelled by specifying a joint distribution of spatial confounders and exposure that allowed for different ranges of spatial correlation and permitted a correlation between U and exposure. To identify the unmeasured confounding bias, Schnell and Papadogeorgou (12) provided a set of assumptions, including the main assumption that the spatial scale of exposure is larger or about the same as the unmeasured confounder (see Additional file 1 for more details).

Both approaches were fit within the Bayesian paradigm, and Gaussian priors were used for regression coefficients with a mean of 0 and standard deviation of 10. Within the Bayesian paradigm, the spatial confounder U was viewed as a missing variable that was iteratively imputed using a Gibbs sampler. Posterior distributions were drawn using 10,000 Gibbs sampler iterations after 1000 burn-in iterations.

The main aim of our statistical analysis was to quantify the causal effect of a one percentage point increase in physician density on late-stage colorectal cancer distribution. We used the most common estimand for continuous treatments, the population average exposure–response curve. The population average exposure–response curve can be interpreted as the posterior distribution of exponentiated exposure coefficient, that is, the relative expected risk of late-stage CRC due to a one percentage point increase in physician density in a randomly chosen municipality. A posterior probability of relative risk lower than 1 indicates a negative association, that is, a protective effect of higher physician density and CRC outcome. The posterior probability of relative risk higher than 1 indicates a positive association, that is, a harmful effect of higher physician density on CRC outcome.

**Fig. 1:**
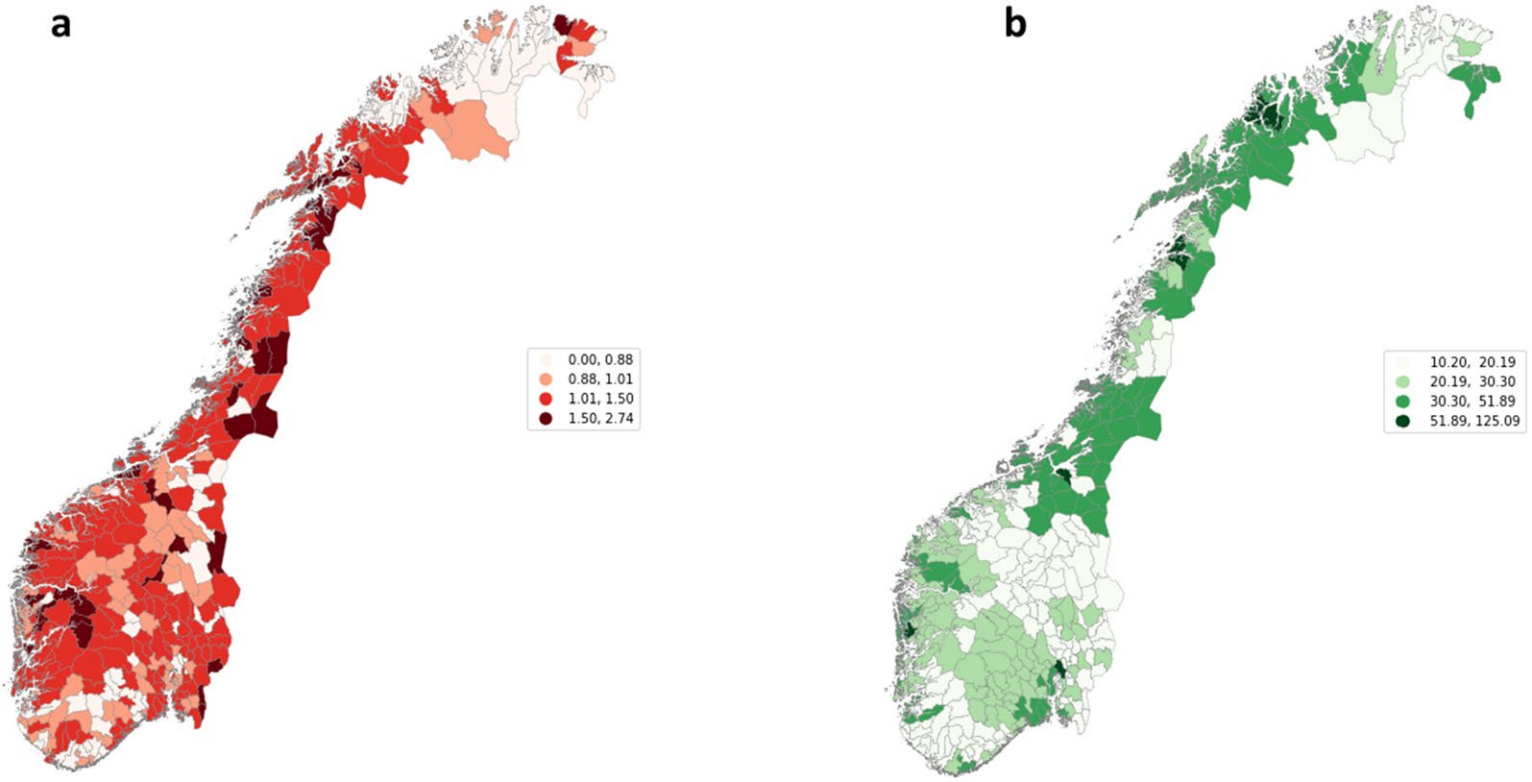
**a.** Municipality-level relative risk rates for late-stage CRC, 2012–2020, Norway (The risk rate is defined as the ratio between the number of observed and expected CRC cases in a municipality). **b.** Municipality-level physician density per 100,000 people, 2011, Norway (Physician density is defined as the number of physicians per 100,000 inhabitants in a municipality)

We used 95% credible intervals to assess statistical significance. The statistical analyses were performed in R software, and the codes for the BYM and NA approaches available from Schnell & Papadogeorgou’s GitHub site (https://github.com/schnellp/causal-spatial) were used and adjusted accordingly Choropleth maps (Fig. 1a and b) were made in Python software (*mapclassify* package) by using the Fisher-Jenks classification method, to determine optimal classes for visual data presentation.

## Results

### Descriptive statistics results

Table 2 shows the total annual number of colorectal cancer cases per 100,000 inhabitants for all stages for the period 2012–2020. The numbers for men and women are generally the same, with late-stage disease having a significantly higher incidence compared to early-stage cases. During the reported period, 583 new cases per 100, 000 inhabitants were diagnosed in late stage, which is 78% of all CRC cases.

**Table 2:** Characteristics of cancer patient population (2012–2020) by stage and sex

Stage of a cancer	Total, n (%)	Men, n (%)	Women, n (%)	Older than 65 years at diagnosis, n (%)
Early stage	7.758 (21.5)	4.076 (21.8)	3.682 (21.2)	5.616 (21.5)
Late stage	28.328 (78.5)	14.623 (78.2)	13.705 (78.8)	20.446 (78.5)
All stages included	36.086 (100)	18.699 (100)	17.387 (100)	26.062 (100)

Figure 1a presents the geographic distribution of the crude relative risk rates for late-stage CRC (adjusted for age and sex), while Fig. 1b presents the number of physicians per 100,000 population across the 356 Norwegian municipalities. Out of all municipalities, 222 have a higher than one crude relative risk rate for the late stage and are mostly accumulated in rural or sparsely populated areas. Figure 1b shows that physician density was unevenly distributed and tends to be higher in urban areas. By inspecting the two maps, a relationship between higher physician density and a lower-than-expected incidence rate could not be anticipated.

**Fig. 2:**
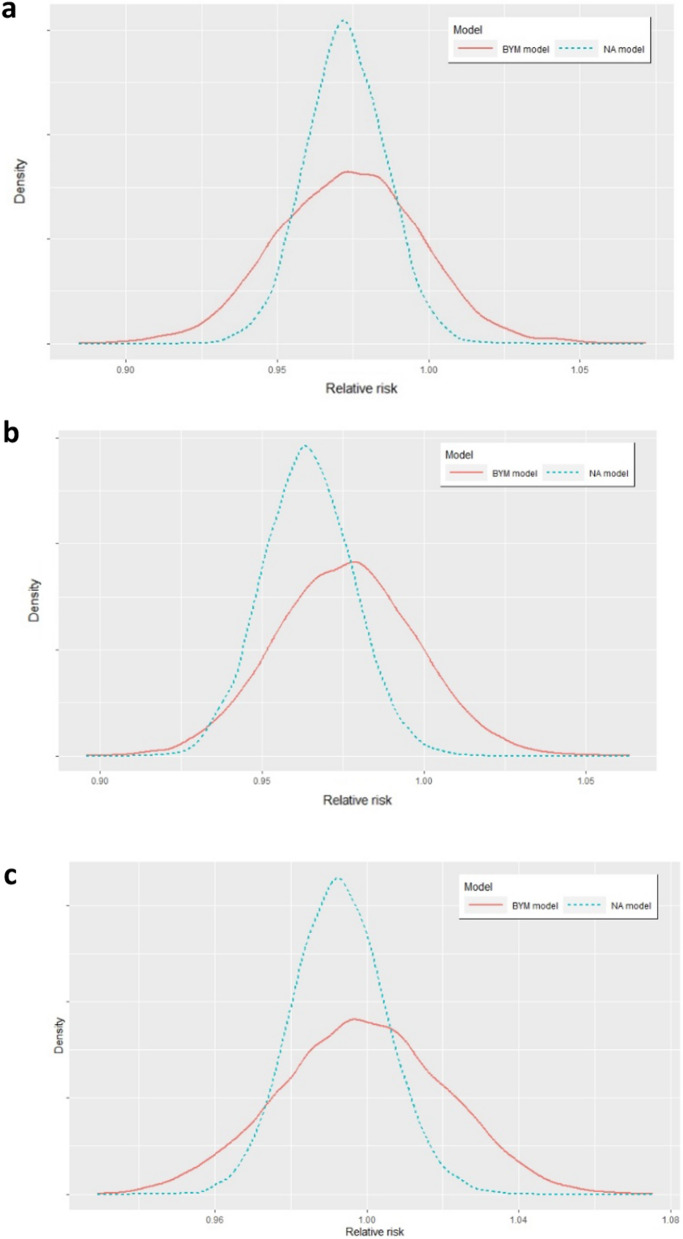
**a.** Model 1 (total physicians’ density), posterior densities of municipality-level relative risk of late-stage CRC. **b.** Posterior densities of municipality-level relative risk of late-stage CRC for Model 2 (GP density). **c.** Posterior densities of municipality-level relative risk of late-stage CRC for Model 3 (specialists’ density)

We calculated the aggregated relative risk rate for late-stage CRC for the municipalities with physician densities within the first quartile (with less than 17 physicians per 100,000 inhabitants). On average, these municipalities had a 9% higher than expected incidence rate for late-stage CRC, or a 1.09 relative risk rate. In contrast, municipalities within the last quartile (with more than 31 physicians per 100,000 inhabitants) had, on average, a 6% lower than expected incidence rate for late-stage CRC, or a 0.94 relative risk rate.

### Statistical analysis results

Table 3 presents the results of the statistical analyses. BYM and NA modelling approaches were performed on the late-stage incidence rates of CRC by specifying three model sets for different exposure variables (see “Statistical analysis” Section).

**Table 3:** Percentage change in late-stage CRC incidence given the infinitesimal change in exposure

Late-stage CRC	BYM approach		NA approach	
	Mean coefficient	Credible interval	Mean coefficient	Credible interval
Model 1 (total physicians)	−0.027	(−0.078 to 0.020)	−**0.028**	**(**−**0.055 to **−**0.001)**
Model 2 (GPs)	−0.025	(−0.070 to 0.018)	−**0.037**	**(**−**0.064 to **−**0.008)**
Model 3 (specialists)	−0.001	(−0.004 to 0.040)	−0.007	(−0.031 to 0.017)

Significant results according to 95% credible interval are shown in bold

According to the NA approach results, the association between total physician density and the incidence rates of late-stage CRC was negative and significant (model 1). Based on the mean coefficient, a one-unit increase in physician density (or an increase of one physician per 10,000 inhabitants) would be equal to the average decrease in late-stage incidence rates by 2.79% (CI −0.055 to −0.001). The corresponding mean coefficient in the BYM approach is nearly identical with the mean coefficient of NA approach, however, it is not significant (CI −0.078 to 0.02). The credible interval for the NA approach indicates that there is a 95% probability that the mean coefficient will lie between −0.055 and −0.001, given the observed data. This means that an increase in the number of physicians in a randomly chosen municipality will, with a 95% probability, result in a lower number of late-stage CRC cases. The corresponding 95% credibility interval for the BYM approach means that an increase in the number of physicians in a randomly chosen municipality can result in a lower but also a higher number of late-stage CRC cases.

When observing GPs and specialists separately, according to the BYM results, there is no evidence of a significant association between GPs and late-stage incidence rates based on the credible interval. However, according to the NA estimation of the mean coefficient, the effect is significant, and a one-unit increase in GP density (or an increase of 1 GP per 10,000 people) will result in on average 3.6%decrease in late-stage rates, ranging from −6.4 to −0.8 percentage change. The difference in the point estimates and belonging CI between models 1 and 2 could indicate the confounding effect of the variable U. For specialists, there is no evidence of a significant correlation with late-stage rates for either modelling approach based on credible intervals.

According to the results for Model 1, the accessibility to both physician groups combined, GPs and specialists, are found important in the early detection of CRC.

Figure 2a shows the posterior distribution of the exponentiated relative risk for NA and BYM estimates, or population average exposure response curve, which can be interpreted as the relative expected risk of late-stage CRC due to a one percentage point increase in physician density in a randomly chosen municipality, as noted in 2.2.

As can be observed from Fig. 2a, the posterior geometric mean of the relative risk for Model 1 was lower than 1 for NA approaches and equal to 0.97 (CI 0.94–0.99). In other words, NA approach reported a protective effect of the higher availability of physicians, that is, a higher availability of total physicians results in a lower expected relative risk of late CRC diagnosis. However, if comparing the 95% CI, for the BYM approach this interval is much wider and ranges between 0.92 and 1.02, which indicates non-significant results. The BYM result implies that the effect can be either protective or harmful.

Figures 2b and 2c show the posterior densities of the municipality-level relative risk of late-stage CRC due to a 1 percentage point increase in GPs (Model 2) and specialist density (Model 3).

For GP density as key exposure, the posterior geometric mean for the NA approach is equal to 0.96 and the CI ranging from 0.93 to 0.98 is indicating a significant protective effect of exposure. For the BYM approach the posterior density suggests a protective effect, but this effect is not significant due to the relatively large share of the distribution with values higher than 1. For specialist density as key exposure, the posterior densities for both approaches have centers slightly below 1, indicating that the results are not significant, or that the exposure can have either a protective or harmful effect.

As shown in figures, the NA distribution is slightly shifted to the left for Models 2 and 3, indicating the presence of the confounding variable U, which is likely to bias the BYM estimator upward. In addition, the NA approach tends to produce more precise results owing to the reduced variance and narrower credible interval compared with the BYM approach.

## Discussion

This study aimed to evaluate regional variations in late-stage CRC diagnosis in association with disparities in physician density. According to the results of the study, areas with a higher GP density have lower-than-expected late-stage incidence rates of CRC. These results confirm previous findings that an increase in GP supply significantly improves CRC outcomes. Additionally, our research showed that a higher density of total physicians (GPs and specialists combined) is negatively associated with late-stage CRC. This implies that accessibility to both physician groups is important in the early detection of CRC, as the GPs recommendation is a critical factor for screening.

Several studies have examined the relationship between physician density and CRC distribution. Ananthakrishnan et al. [8] found that primary care physician density and gastroenterologist density were inversely correlated with the county-level incidence of late-stage CRC. However, this study did not find an association with overall physician density and this relationship was only true for rural counties or counties with low population density. Roetzheim et al. [4] found that higher PCP is negatively correlated with CRC incidence and mortality, as well as with late-stage CRC diagnosis. These authors did not find a relationship between gastroenterologist density and late-stage CRC.

Our results are consistent with the findings of these studies, when considering the densities of GPs and specialists separately. However, while previous literature explored the association between specialists and CRC outcome by including only the number of disposable gastroenterologists, we included other relevant specialists, such as surgeons and diagnostic pathologists. Also, in contrast to previous research, our results shows that an overall increase in physicians (Model 1, GPs and specialists combined) was significantly associated with lower late-stage diagnosis. Specifically, GPs play a significant role in advising patients and recommending screening which is essential for CRC detection in the early phase. However, there are several barriers to physicians’ recommendations regarding CRC screening. Lack of time and heavier workload could affect GP decisions, as the discussion about colonoscopy takes more time than discussions about other cancer screening tests [1]. In addition, barriers to CRC screening may be related to long delays in colonoscopy scheduling and a lack of direct access to colonoscopies [1, 3]. For example, if GPs are faced with a reduced workforce in local hospitals and challenges in colonoscopy scheduling, they may adopt a higher threshold before sending referrals to hospitals or choose rapid tests (e.g. barium enema tests) to avoid long delays [1, 6]. Therefore, the study emphasise that accessibility to physician groups, GPs and specialists, is essential for improving the overall CRC outcome.

To our knowledge, this is the first study to evaluate the effect of physician density on cancer outcomes within a causal inference framework. This is the first application of the NA approach to this subject. Compared with the BYM approach, which did not find a significant effect of physician density on late-stage CRC cases or report even null or harmful effects, the NA approach provided results that are more credible and in line with subject-matter knowledge. Higher physician density is likely to have a protective effect, that is, people living in such areas are less likely to have late-stage CRC diagnoses. The NA approach tends to produce more precise results due to reduced variance compared to the BYM approach and tends to shift the distribution slightly to the left, indicating that unobserved spatial confounding is likely to bias the BYM estimatesupward. Different spatial confounders could affect both the exposure and the outcome of interest. For example, working conditions of health workers, substandard medical equipment and facilities, quality of training and educational systems, or other place-specific environmental conditions could affect physician density, but also the distribution of late-stage CRC [16, 11]. It might be less appealing for physicians to place their offices in rural areas with fewer opportunities and poorer working conditions. This factor could also be a predictor of higher late-stage CRC incidence rates because of lower healthcare quality and potential increases in medical errors.

The main limitation of the study is that it was conducted at the municipality-level, precluding inferences at the individual level. Although estimating the effect at the municipality level could help inform policymaking at local and regional levels, a multilevel approach that includes individual, social, structural, and spatial levels of influence is also important when dealing with research on cancer prevention policies [16]. A second limitation is the lack of availability of relevant individual-level risk factors aggregated to the municipality level, such as the fraction of inhabitants with obesity. Additionally, some assumptions were made to draw causal conclusions. One of the key assumptions was that the spatial scale of the confounder is larger than that of the exposure [12]. This means that not all confounders with a smaller spatial scale will be mitigated.

This study points out that better healthcare accessibility, particularly physician availability, could impacts disease prevention and potential health gains at the regional level. For many years, Norway has confronted challenges in attracting, recruiting, and retaining physicians in rural areas. This study highlighted the importance of overcoming these challenges and shaping policies for geographically equal access.

## Conclusion

In conclusion, this study showed that an increase in the availability of physicians improves CRC outcomes. The findings highlight the importance of the right composition of physician workforce, which could also be worth further research. Increasing the number of specialists in gastrointestinal diagnostic and preoperative investigations without an increase in PCP, will not significantly improve cancer outcomes.

This study has important implications for decision-making at the local and regional levels. This suggests that policies to improve healthcare accessibility should target areas with lower physician-to-population ratios to ensure equal geographic accessibility and accelerate the early detection of disease. To fully inform public health authorities and clinical practices, and determine which intervention strategy to introduce, more comprehensive research on the feasibility, efficacy, and economic consequences of these policies is required.

The study has particularly accentuated the efficiency of the NA approach in investigating the relationship between physician density and late-stage CRC in the presence of unmeasured spatial confounders, with estimates that are qualitatively diverse from the classical model estimates and more in line with subject-matter knowledge. We expect this methodology to be replicable in other countries and for other types of preventable diseases, where physician support is important for their early detection. This is especially true for countries with registry systems that do not allow for easy matching of administrative and health-related registries.

## Supplementary Information

**Additional file 1. ** Additional information on neighbourhood adjustment via spatial smoothing methodology.

## Data Availability

Raw data were generated from Cancer Registry of Norway. Derived data supporting the findings of this study are available from the corresponding author DD on request. The data are not publicly available due to restrictions e.g., their containing information that could compromise the privacy of research participants.
