# Supplementary material for: The effect of physician density on colorectal cancer stage at diagnosis: causal inference methods for spatial data applied on regional-level data
Source: Int J Health Geogr. 2023 Jan 19;22:1. doi: 10.1186/s12942-023-00323-w (PMC9850813; doi:10.1186/s12942-023-00323-w)
Supplement: Supplementary file 1 — Additional file 1. Additional information on neighbourhood adjustment via spatial smoothing methodology. [file 12942_2023_323_MOESM1_ESM.pdf]

ADDITIONAL FILE 1

Additional information on neighbourhood adjustment via spatial smoothing methodology

A spatial neighbourhood adjustment method was developed with the aim of directly adjusting for spatial confounders to provide more accurate effect estimates and strengthen the causal claim of scientific conclusions (Schnell & Papadogeorgou, 2020). The main assumption of this approach is that neighbouring areas will have the same values of such confounders.

The authors of the model started with the claim that the commonly used approach to estimate $\hat{\beta}$ is biased in the presence of u_i and that this bias is the result of non-zero correlation between the confounder and exposure, which leads to a non-zero conditional expectation E(u_i|Z) (Schnell & Papadogeorgou, 2020).

The main aim of this method is to remove spatial trends from the outcome, exposure, or both by joint modelling of the exposure and the missing spatial confounder:

$$Y_i = \beta Z_i - B(X) + \gamma C_i + \varepsilon_i \quad (1)$$

where $B(X) =$ E(u_i|X) is the bias term and X is the design matrix containing the intercept, exposure, and measured covariates. As the bias term E(u_i|X) cannot be calculated directly, as the u_i is unobserved, Schnell and Papadogeorgou provide a set of assumptions that will assist in estimating this term. The assumptions are based on the Gaussian Markov random field for the joint modelling of the $(u_i, Z_i)|C_i$. They assume that u_i and ε_i are Gaussian with mean zero, $(u_i, Z_i)|C_i$ is multivariate normal and independent of C_i.

In addition, they set two additional assumptions: a cross-Markov property and a constant conditional correlation. The cross-Markov property states that the local exposure Z_i at location i is correlated only with the local confounder u_i and does not allow u_j at location j to directly affect the value of Z_i. The constant conditional correlation assumption assumes that the conditional correlation between u_i and Z_i does not vary with location. The model is also based on standard causal inference assumptions: temporal order, stable unit treatment values, and positivity assumptions (Rubin, 1975).

In the joint distribution of the $(u_i, Z_i)|C_i$ these assumptions can be incorporated into the precision matrix

$$\binom{u_i}{Z_i} \sim N\left[\binom{0}{C_i\gamma}, \begin{pmatrix} G & Q \\ Q^T & H \end{pmatrix}\right] \quad (2)$$

where G and H are the precision matrices of $u_i|(Z, C_i)$ and $Z|(u_i, C_i)$ (Schnell & Papadogeorgou, 2020).

The Restricted Maximum Likelihood framework allowed us to investigate bias by integrating the distribution of unmeasured confounders from the observed data likelihood. However, as REML is not applicable for continuous treatments, the authors extended the estimator to a non-linear model within the Bayesian implementation. Inference was conducted within a Bayesian framework by fitting the model using a Markov chain Monte Carlo algorithm and iteratively imputing the missing variable u_i through a Gibbs sampler.

Literature:

Rubin, D. B. (1975). Estimating causal effects of treatments in randomized and nonrandomized studies. *Journal of Educational Psychology*, *66*(5), 688. https://doi.org/10.1037/h0037350

Schnell, P. M., & Papadogeorgou, G. (2020). Mitigating unobserved spatial confounding when estimating the effect of supermarket access on cardiovascular disease deaths. *The Annals of Applied Statistics*, *14*(4), 2069–2095. https://doi.org/10.1214/20-AOAS1377
